# Metabolic Bone Disease in Captive Flying Foxes: A Comprehensive Survey Across Zoological Parks

**DOI:** 10.3390/vetsci12030271

**Published:** 2025-03-13

**Authors:** Diana Faim, Filipe Silva, Anton Weissenbacher, Iris Starnberger, Isabel Pires

**Affiliations:** 1 Department of Veterinary Sciences, School of Agricultural and Veterinary Sciences (ECAV), University of Trás-os-Montes e Alto Douro, 5000-801 Vila Real, Portugal; fsilva@utad.pt (F.S.); ipires@utad.pt (I.P.); 2 Onevetgroup Hospital Veterinário de Leiria (HVL), 2410-270 Leiria, Portugal; 3 Centre for Animal Sciences and Veterinary Studies (CECAV), Associate Laboratory of Animal and Veterinary Sciences—AL4AnimalS, University of Trás-os-Montes e Alto Douro, 5000-801 Vila Real, Portugal; 4 Vienna Zoo (Tiergarten Schönbrunn), 1130 Wien, Austria; a.weissenbacher@zoovienna.at (A.W.); i.starnberger@zoovienna.at (I.S.)

**Keywords:** flying foxes, metabolic bone disease, vitamin D3, diet

## Abstract

Metabolic bone disease (MBD) is clinically characterized by bone deformities and is associated with vitamin D3 deficiency in diurnal animals. However, the pathogenesis and etiology of this condition in flying foxes, considered nocturnal animals, are poorly understood. Therefore, we conducted a survey aimed at various zoological parks housing flying foxes to elucidate the pathogenesis and etiology of MBD in these animals. Our results indicate that vitamin D3 may play a role in preventing metabolic bone disease in flying foxes due to its involvement in calcium absorption. However, these nocturnal animals seem to obtain vitamin D3 primarily through dietary sources in contrast to the cutaneous absorption described in diurnal species. Additionally, our results suggest that an appropriate diet for this species, including fruits, green vegetables, and other protein sources such as animal products and mineral supplementation, could contribute to preventing metabolic bone disease.

## Simple Summary

Flying foxes, also known as fruit bats, can suffer from metabolic bone disease (MBD), a condition that causes bone deformities and is linked to a lack of vitamin D3. Unlike some other animals, flying foxes are thought to obtain most of their vitamin D3 from their diet rather than from sunlight. This study surveyed different zoos to understand why MBD occurs in these nocturnal animals. The results show that feeding flying foxes a diet rich in vitamin D3 is essential to prevent the disease. These findings highlight the importance of proper nutrition for these bats in captivity and could help improve their care and conservation.

## 1. Introduction

Flying foxes (genus *Pteropus* spp.) are a genus of bats within the order Chiroptera, which includes over 60 species worldwide [1]. Flying foxes are distributed across three continents, extending from the eastern Mediterranean to the western Pacific [2]. According to the International Union for the Conservation of Nature, most species in the genus *Pteropus* are listed as vulnerable or endangered [3].

Flying foxes, the largest known bats, can have a wingspan up to 1.5 m and a head and body length of approximately 0.4 m. Bat wings are formed by a flexible membrane that extends from the abdomen to the tips of their digits, encompassing the entire skeletal system known as the patagium [4,5].

Flying foxes primarily feed on fruit and flower products, such as pollen and nectar [6,7]. They also consume tree leaves, extracting only their sap through a process known as leaf fractionation [6,8]. Although they are frugivorous and nectarivorous, insects, which are extremely rich in protein, can also become a part of their diet when less fruit and vegetation are available during the dry season [9,10].

These animals typically inhabit forests in tropical or subtropical regions [7] and organize themselves into colonies. Although they are social animals, dominance phenomena are common, especially in captivity, where space is sometimes limited [11]. Therefore, it is essential for zoological parks to provide spacious environments that promote continuous flight and the species’ natural behaviors [12]. Vertical perches and multiple resting and feeding areas are particularly important due to the social hierarchy in which they are organized. Introducing elements that mimic their natural habitat also enhances their social interactions and overall well-being. Examples include the creation of refuges with shrubs, climbing branches, or hanging objects [12,13]. Environmental enrichment significantly impacts health by reducing stress, abnormal behaviors, and dominance-related actions, which can lead to adverse health effects in flying foxes [11,14].

Metabolic bone disease (MBD) is a group of disorders influenced by dietary and non-dietary factors, including conditions such as rickets, osteomalacia, and fibrous osteodystrophy. Rickets and osteomalacia are both characterized by a failure of bone mineralization, leading to bone deformities and fractures [15]. Osteomalacia occurs in adult animals, while rickets affect young animals, specifically targeting the growing skeleton [16]. In rickets, there is also hypertrophic thickening of the zone of the growth plate, where improper endochondral ossification occurs. The most common underlying causes of both conditions are deficiencies in essential nutrients, particularly vitamin D or phosphorus [15]. Fibrous osteodystrophy results from both primary and secondary hyperparathyroidism (whether nutritional or renal) and is characterized by reduced bone mass and increased bone flexibility due to the resorption of bone and its replacement by fibro-osseous tissue. This process significantly weakens the skeletal structure, making it prone to fractures and deformities [17]. Primary hyperparathyroidism typically occurs due to a functional adenoma in the parathyroid gland, leading to the overproduction of parathyroid hormone (PTH) [17]. Secondary hyperparathyroidism can be nutritional, resulting from diets low in calcium and high in phosphorus, and is most common in young, growing animals fed diets deficient in calcium with a relative excess of phosphorus [15]. It can also be renal, where kidney failure causes decreased phosphorus secretion and reduces the synthesis of 1,25-dihydroxyvitamin D, disrupting the balance of calcium and phosphorus in the body [18].

Clinical signs suggestive of metabolic bone disease, such as long bones and jaw deformities [15], have recently been observed in flying foxes in some zoological parks (personal observation). Metabolic bone disease (MBD) is characterized by extreme bone remodeling, leading to calcium depletion at the bone level or hypomineralization [16]. It has a high incidence in reptiles due to improper handling and feeding practices [19,20,21] and has been reported in some bat species but not in flying foxes [22,23,24,25]. The disease has been described among little brown bats (*Myotis lucifugus*) during periods of calcium stress, which coincide with hibernation, where there is no calcium intake, and during pregnancy, when calcium demands are higher. It has also been described in *Myotis lucifugus* and *Miniopterus schreibersii* (Schreiber’s long-fingered bat) during lactation [26,27] and in bats weaned prematurely, as observed in the big brown bat (*Eptesicus fuscus*) [28,29]. The most frequently reported signs are bone deformity, leading to wing arching, joint inflammation, fractures, reluctance to move, and inability to fly [28,29]. In cases of prolonged deficiency, they may also present with neuromuscular hyperirritability, tetanic spasms, and seizures [29]. Other cases were described in the common vampire bat (*Desmodus rotundus*) for two juveniles with rickets, although the cause was undetermined [30]. The assumption that calcium is regulated according to its physiological needs and is independent of the presence of vitamin D3 [23,27] is underpinned by the aforementioned examples and by the literature found in a broad survey.

To our knowledge, there are no published cases of MBD in flying foxes (*Pteropus* spp.). While flying foxes are a type of bat and are classified as nocturnal animals, they have significantly different daily habits compared to other bat species, which may influence the development of metabolic bone disease. Unlike other bat species, which are not exposed to sunlight and therefore cannot produce vitamin D3 endogenously, flying foxes rest in tree canopies where they are exposed to light [11].

This peculiarity raises the question of whether flying foxes, despite being nocturnal, have undergone the same evolution as other bats regarding vitamin D3 production and calcium absorption mechanisms.

This study aims to determine the global prevalence of metabolic bone disease in flying foxes and to discuss whether feeding practices similar to those in diurnal species might be associated with this condition. Additionally, we assess whether enclosure conditions—such as space availability (considering their role in social hierarchy and resource access), temperature, and access to natural light—influence disease prevalence.

## 2. Materials and Methods

### 2.1. Survey Development

The questionnaire was developed based on a literature review considering the study’s objectives. It was submitted and approved by the Ethics Committee (TGS 2022/1016) and the veterinary team of Tiergarten Schönbrunn (Vienna Zoo, Wien, Austria).

The questions were set up in Google Forms and consisted of 29 short-answer, multiple-choice, and selection questions to ensure clarity and avoid misinterpretations or data omission. The content is divided into three main parts: diet, enclosure conditions, and health status. The survey includes questions on the zoological park, diet and nutrition, facilities, and health status. The section on zoological park details collects basic information about the name and geographic location of the park, as well as the flying fox species housed there. In the diet and nutrition section, we gather information regarding the types of food provided to the animals, including fruits and vegetables, nectar, pollen, animal products, and other food. We ask for details on specific food types and proportions, whether supplements are used, and, if so, what kinds and quantities are provided.

Additionally, we inquire whether a structured nutritional plan or menu is followed. The facilities section focuses on the living conditions of the flying foxes. It includes questions about the space allocated per animal, the type of enclosure (indoor, outdoor, or both), the temperature within the enclosures, and access to natural and artificial light sources. The final section inquires if the park has flying foxes with signs indicative of metabolic bone disease and, if so, asks about the number of animals, their age, sex, pregnancy status, medical history, and any organ dysfunction.

The complete survey is available in the Supplementary Files.

### 2.2. Participant Recruitment and Survey Distribution

Zoological parks included in this study were selected based on their exclusive housing of flying foxes (*Pteropus* spp.), ensuring consistency in management conditions, particularly feeding practices. The study targeted all zoological institutions housing flying foxes globally, with participant recruitment conducted using the Zoological Information Management System (ZIMS) [31]. Additionally, research was performed on the official websites of zoological institutions to confirm eligibility.

A total of 51 zoological parks were chosen as candidates, and their contacts were obtained via a Microsoft Excel document from EAZWV (European Association of Zoo and Wildlife Veterinarians). The survey was distributed via email every three weeks for four months, starting on 24 January 2023.

### 2.3. Data Processing

The statistical analysis was conducted using SPSS version 23 (Statistical Package for the Social Sciences, IBM Corp^®^, Armonk, NY, USA). Data were analyzed in two main steps: a descriptive analysis of all the animals and of the animals with metabolic bone disease followed by comparative statistical tests to assess potential associations between diet, enclosure conditions, and the presence of metabolic bone disease (MBD).

#### 2.3.1. Descriptive Analysis

Given the open-response nature and variability of the questionnaire, some data were categorized and restructured without altering their content or accuracy to facilitate interpretation. The main dietary categories included the color of fruits and vegetables, classified into five groups: red, orange/yellow, purple/blue, white, and green. The proportion of each color group in the diet was then calculated, considering the fruits mentioned by the parks.

Responses regarding animal products were categorized as Yes/No. The “other food” category encompassed non-fruit and non-vegetable items, nectar, and animal products, which were grouped into four subcategories: cereal (oat flakes), frugivorous bird food, dog food, and primate food.

Regarding enclosure conditions, the space provided was classified as ideal or non-ideal, following each species’ AZA Bat TAG guidelines. Enclosures were considered ideal when their dimensions corresponded to at least four times the wingspan in both length and width for the housed flying fox species [29]. Similarly, temperature conditions were categorized as ideal or non-ideal, based on optimal values described in the literature, ranging from 291.15 K to 305.15 K [13,30,31].

All the remaining questions were categorized as ‘Yes’ or ‘No’.

#### 2.3.2. Statistical Comparative Analysis of Symptomatic and Healthy Groups

Statistical tests were applied to compare differences between flying foxes with clinical signs of metabolic bone disease (MBD) and those considered healthy (i.e., without clinical signs). Statistical significance was assessed using Fisher’s exact test and Pearson’s chi-square test for categorical variables (e.g., Yes/No responses related to diet, facility conditions, and management practices). Given the small sample size and the potential for some expected frequencies to fall below 5, a continuity correction was applied to the chi-square test.

For continuous variables (e.g., the percentage of specific fruit colors consumed), data distribution was first evaluated using the Kolmogorov–Smirnov test to check for normality. Since the data did not follow a normal distribution, comparisons were performed using the Mann–Whitney U test for independent samples (non-parametric).

The total number of animals per zoo was calculated using the ZIMS (Zoological Information Management System by Species360) [31], an electronic data management tool for global zoo and aquarium collections.

## 3. Results

### 3.1. General Descriptive Analysis

#### 3.1.1. Zoological Parks: Location and Species

A total of 51 zoological parks were initially contacted, but only 13 institutions responded and agreed to participate in the study (25.49%), encompassing 538 individuals. One institution was excluded due to unknown animal numbers and because it is a cub recovery center with different objectives and methodologies than a zoo. Due to data protection policies and the agreements established with the participating zoological parks, specific details regarding each institution cannot be disclosed.

**Figure 1:**
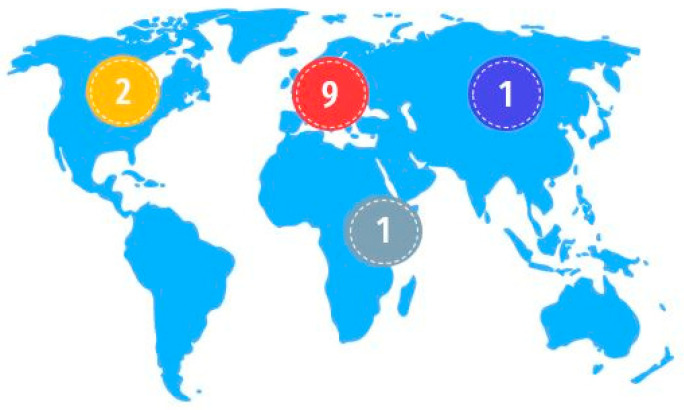
Distribution and number of zoological parks. World map adapted from Slidesgo (Freepik Company, S.L.U., Málaga, Spain).

The study encompasses representation from all continents except Antarctica and Oceania, with the largest portion corresponding to Europe, represented by nine zoological parks housing 368 animals. In America, there are two parks with 150 individuals, while Africa and Asia have one park with 12 and 8 flying foxes, respectively. Figure 1 demonstrates the geographical distribution of the participants.

In Europe, the species present in the parks include *Pteropus lylei*, *P. giganteus*, *P. vampyrus*, *P. rodricensis*, and *P. livingstonii*. In Asia and Africa, *Pteropus vampyrus* is the only species present. In America, a more diverse range of species is found, including *P. poliocephalus*, *P. pumilus*, *P. giganteus*, *P. vampyrus*, *P. rodricensis*, *P. conspicillatus*, and *P. hypomelanus*. It is important to note that this distribution does not reflect the species’ natural habitats, but rather the species present in these institutions.

**Figure 2:**
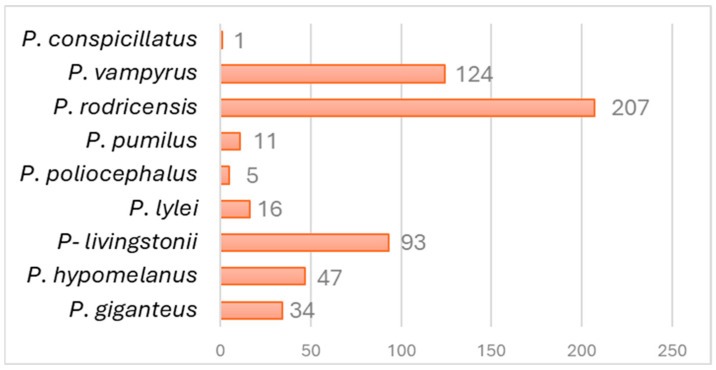
Number of individuals per species in the zoological parks studied.

As shown in Figure 2, the species with the highest representation are the Rodrigues flying fox (*Pteropus rodricensis*, *n* = 207), the large flying fox (*P. vampyrus*, *n* = 124), and the Comoro flying fox (*P. livingstonii*, *n* = 93).

#### 3.1.2. Diet

Regarding diet, all the parks, except for one, follow a strict food plan to ensure consistency among all the animal keepers.

- Fruit and vegetables

**Figure 3:**
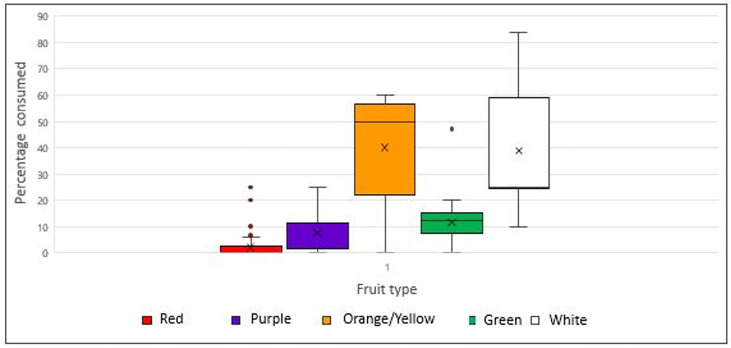
Percentage of fruit per color group consumed by flying foxes in the participating parks (x: mean, •: outliers).

The surveyed parks’ staple diet consists of various fruits and vegetables. As shown in Figure 3, the animals predominantly consume orange/yellow fruits (median: 50.0%, range: 0–60%) and white fruits (median: 25.0%, range: 10–83.8%). In contrast, the consumption of green fruits and vegetables is lower (median: 12.5%, range: 0–47.2%), followed by purple/blue fruits (median: 11.5%, range: 0–25%).

- Animal products and other types of food used

In addition to fruit, which has low protein content, other protein sources were mentioned in the feeding of flying foxes (Table 1), such as nectar, consumed by 6.9% of our sample (*n* = 37), animal products such as cottage cheese, yogurt, eggs, and honey with a percentage of 5.4% (*n* = 29), and other food items present in the diet of 62.3% of the flying foxes (*n* = 335). Notably, 297 animals regularly consume primate food (55%). Table 1 describes the other foods provided to the flying foxes included in the study.

- Use of nutritional supplements

Zoological parks also use supplements to complement the minerals and vitamins absent or insufficient in fruits; however, this applies to less than half of the cases (41% of flying foxes, *n* = 218). Various supplements were described in our survey of the zoos, such as brewer’s yeast (*n* = 23), mineral blocks (*n* = 145), vitamin E (*n* = 8), vitamin D3 (*n* = 23), calcium (*n* = 58), and several multivitamins. These include supplements formulated for birds (*n* = 29), supplements for human use (*n* = 39), and supplements for flying foxes (*n* = 150), each used by two parks.

#### 3.1.3. Flying Fox Enclosures

- Enclosure dimensions (space per animal)

The enclosure sizes provided by each zoological park were analyzed and classified based on their compliance with AZA Bat TAG guidelines and species-specific requirements [29] as described in the Section 2.

Based on the submitted responses and analysis of the appropriate space for each species, 58% of the animals (*n* = 313) are housed in suitable areas in nine parks.

- Enclosure temperature

Similar to the number of square meters per animal, the temperature range specified by each location was also classified as ideal or non-ideal. All parks have an ideal temperature (291.15 K and 305.15 K), except for two that exceed the upper limit (308.15 K and 313.15 K). The number of flying foxes (*n* = 35) subjected to these temperatures amounts to 7% of all animals surveyed in this study.

- Access to natural/artificial light (UVB radiation)

All zoological parks have indoor spaces (enclosed areas), but three provide additional access to outdoor areas with direct access to sunlight or natural light without barriers (e.g., glass or plastic ceilings). Thus, in these three zoos, flying foxes (*n* = 173; 32%) can access UVB radiation through the sun. Three parks provide artificial light through ultraviolet lamps for 46 flying foxes (9%) all year round. Fifty-nine percent of the animals cannot access natural or artificial UVB radiation.

**Figure 4:**
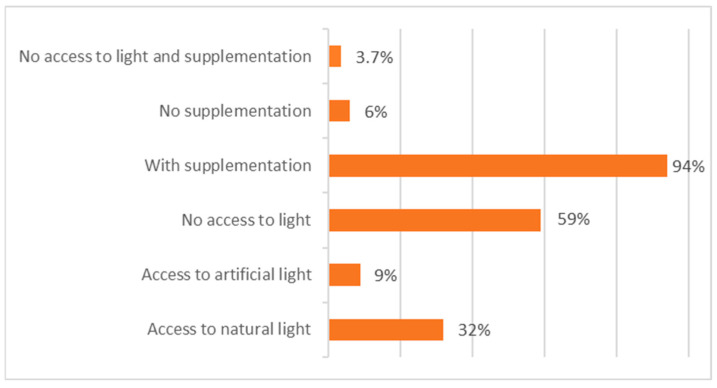
Number of flying foxes with access to different light conditions and vitamin D3 supplementation.

Considering the potential production of and access to vitamin D3, either through exposure to UVB radiation (available to 41% of the animals) or through dietary supplements (provided to 94% of the animals), as shown in Figure 4, it is noteworthy that only 3.7% (*n* = 20) of our sample neither have access to UVB radiation nor receive any vitamin D3 supplementation.

### 3.2. Descriptive Analysis of Metabolic Bone Disease Animals

#### 3.2.1. Animals Affected

Two zoological parks have described cases compatible with metabolic bone disease. In the first park, which houses twenty-three animals, six individuals exhibit bone changes suggestive of metabolic bone disease (26% of the population): five females and one male. In the second park, three clinically affected animals are found in twelve individuals (25% of the population). We don’t have specific information about the gender of symptomatic animals in the second park, but 75% of the total population is female.

In both parks, all affected animals are large flying foxes (*Pteropus vampyrus*).

No detectable disease was noted in the progenitors, and there were no problems during birth. The animals were raised naturally by their mothers and have lived in the same park since birth.

Both parks reported signs such as deformities of the long bones in the forearm region (bending and arching of the wings) and deformation and flexibility of the jaw. One park also reported exacerbated edema, swelling of the joints, and reluctance to move. In addition to these signs, none of the animals (*n* = 9) can fly due to weakness and deformities.

In all flying foxes, the signs began to develop in the first months of life and have persisted to the present, which may indicate a growth disease (affecting immature bones). Currently, in one of the parks, the six sick animals are adults, between two and fifteen years old. In the second park, two of the three animals were euthanized at one year of age, and the other is currently one year old.

Given the age of symptom onset, none of the animals were pregnant or lactating, and none of the animals with bone alterations were used for breeding.

At birth, none of the animals showed signs similar to those described, potentially ruling out a hereditary disease. However, in the zoo with three affected individuals, there has already been interbreeding among their progenitors, indicating shared kinship. Although genetic tests have not confirmed this, the possibility of inbreeding among individuals is strong. In the other zoo, all affected animals underwent genetic testing, and none had familial relationships.

According to the clinical signs, the affected animals also do not show evidence of dysfunction in other organs, apart from those described above, particularly in the intestines, liver, kidneys, and parathyroid.

#### 3.2.2. Diet

In zoological park 1, the provided diet includes a variety of fruits and vegetables, with more than half of the rations consisting of white fruits (51.87%), followed by orange/yellow fruits (37.03%), and smaller amounts of red (3.7%), purple/blue (3.7%), and green (3.7%) fruits. In addition to fruits and vegetables, the diet includes nectar and animal products, such as cottage cheese, yogurt, and raw eggs. No other food items were included. The park ensures supplementation with calcium, vitamin D3, yeast, and human supplements, but not with supplements designed explicitly for flying foxes.

In zoological park 2, the provided diet also includes various fruits and vegetables, mostly orange/yellow fruits (44.5%), followed by white fruits (33.3%), and smaller amounts of red (11.1%) and purple/blue (11.1%) fruits. Green fruits are not included in the diet. Unlike other parks, zoological park 2 does not provide nectar or animal products such as cottage cheese, yogurt, or raw eggs, nor does it include other food items. Supplementation at the park is limited, with only calcium supplements provided.

Both parks follow a strict food plan.

#### 3.2.3. Enclosures

Regarding enclosures, in zoological park 1, there is no access to natural light, but artificial lighting is used in indoor spaces. However, the enclosure area was not ideal, and it was reported that juveniles (<1 year) were denied access to the lamps and other resources, such as food, by older and more dominant individuals. The indoor temperature range (291.15–313.15 Kelvin) is not ideal.

Zoological park 2 offers an area of recommended size for the species. No artificial lighting is used indoors, and the park does not provide access to outdoor areas with natural light. The indoor temperature range (291.15–308.15 Kelvin) is not ideal. Inbreeding is documented in this park.

Table 2 summarizes the characteristics of the animals and the conditions in zoological parks 1 and 2.

### 3.3. Statistical Analysis of Symptomatic and Healthy Groups

To examine potential statistical associations between dietary intake, enclosure conditions, and the clinical presentation of metabolic bone disease (MBD), two groups were defined: the symptomatic group, including individuals with clinical presentation of the disease (n1 = 9), and the healthy group, without signs of metabolic bone disease (n2 = 503). Animals without apparent disease but housed in parks where affected individuals were present were excluded from the analysis to minimize confounding factors.

Green vegetable consumption was significantly lower in the symptomatic group, with a median intake of 3.7%, compared to 12.5% in the healthy group (*p* < 0.001). Although differences in fruit color consumption were not statistically significant, some results should be noted. The median intake of purple fruit was higher in the healthy group (11.5%) compared to the symptomatic group (3.7%), while orange fruit intake was also higher in the healthy group (50%) than in the symptomatic group (37.03%).

Primate food consumption also was associated with MBD status, as 59% of healthy animals consumed primate food, whereas none of the affected individuals had access to it (*p* = 0.001). Regarding supplement intake, all symptomatic animals received dietary supplements, whereas only 37.6% of healthy animals were supplemented (*p* < 0.001). Among the symptomatic group, human supplements, calcium, vitamin D3, and brewer’s yeast were provided by one zoo. Compared to the healthy group, the intake of these supplements varied between 0% and 4.6%, indicating that supplementation alone does not necessarily prevent MBD. Other dietary variables did not show significant differences; however, none of the affected animals received mineral blocks, whereas 71.2% of the healthy group had access to them.

Among the environmental variables analyzed, all symptomatic animals lacked access to natural light, whereas the majority (65.6%) of the healthy group had access to natural light (*p* = 0.03). In contrast, artificial light seems to not prevent the occurrence of clinical signs, since the majority of affected individuals were exposed to artificial light (*p* < 0.001).

## 4. Discussion

The investigation of diseases affecting captive animals has garnered significant interest within the scientific community, which has led to an improved understanding of the affected species in both captive and wild settings, leading to improved nutrition, management, welfare, and survival rates. Metabolic bone disease (MBD), characterized by bone deformities, is frequently associated with vitamin D3 deficiency in diurnal species [16,19]. However, this condition has also been observed in flying foxes, which are primarily nocturnal, leaving several uncertainties regarding its etiology and manifestation in these animals. To address these concerns, we conducted a survey across multiple zoological parks housing flying foxes. Given that nutritional imbalances caused by inconsistent or inadequate feeding practices can contribute to metabolic bone disease [11,32], and that environmental conditions may also play an indirect role in its occurrence [11,14], the survey included questions on diet, habitat conditions, and the incidence of metabolic bone disease in these facilities.

Only 25.49% of the surveyed zoological parks responded, which represents a major limitation of this study. While the collected data could provide valuable insights into managing flying foxes in captivity, the limited sample size necessitates caution when generalizing the findings. The results should be interpreted as an initial exploration of potential risk factors for metabolic bone disease (MBD) rather than definitive conclusions. The 13 parks with 538 reported flying foxes were distributed on all continents except Antarctica and Oceania, with the majority housed in Europe. Most of the animals included in this study were Rodrigues flying foxes (*Pteropus rodricensis*), followed by large flying foxes (*P. vampyrus*) and Comoro flying foxes (*P. livingstonii*).

As expected for these frugivorous species [6], all parks included in our study provided a fruit-based diet with varying proportions and, in most cases, adhered to a structured nutritional plan. The animals primarily consumed more orange-colored fruits and white fruits and fewer red fruits, purple fruits, and green fruits and vegetables. Fruits and vegetables are excellent sources of essential minerals and vitamins crucial for bone health, including calcium, phosphorus, magnesium, vitamin C, and vitamin D [6,15]. Among them, purple fruits and green vegetables contain the highest calcium levels, while orange-colored citrus and red-category fruits also contribute significant amounts. Optimal phosphorus concentrations are primarily found in purple and red fruits and green vegetables. These food sources generally maintain an ideal calcium-to-phosphorus (Ca:P) ratio of 2:1 or 1:1, which is important for bone metabolism [6,15,33]. Magnesium is predominantly found in purple fruits, green vegetables, and some white and orange fruits, whereas vitamin C, an important factor in collagen synthesis and bone maintenance, is most abundant in green and orange fruits, followed by red fruits. Vitamin D3 is absent from plant-based foods, as it is exclusively found in animal-derived products. Additionally, white fruits provide significant amounts of magnesium, further supporting skeletal health.

Given these nutritional profiles, ensuring a diverse intake of fruits and vegetables can help meet the dietary needs essential for maintaining strong and healthy bones [33]. While the ideal diet for captive flying foxes should mirror their natural feeding habits, obtaining native sweet and soft fruits poses logistical challenges for zoological institutions and rehabilitation centers. The American Zoo and Aquarium Association Chiropteran Taxon Advisory Group offers guidelines on fruit bat diets’ nutrient and mineral requirements. However, additional nutrient intake and metabolism data are needed to refine these recommendations and assess their suitability for different species [7]. Consequently, commercially available fruits and vegetables are often used as substitutes, potentially altering the nutritional composition compared to wild diets. This discrepancy should be considered when evaluating captive feeding strategies’ nutritional and behavioral impacts.

Since fruits have low protein content [6,34], other foods such as pollen and nectar from flowers were used for 37 animals (across three parks) to balance protein levels [11,35]. Animal products, such as cottage cheese, yogurt, eggs, and honey, were used in two parks with 29 animals. Primate food stands out among the foods considered as complete and is consumed by 297 animals (55% of the sample). Although formulated for primates, this food is considered suitable for flying foxes as a supplement to fruit, which is low in protein and certain minerals [6].

Some zoological parks also use supplements to complement the minerals and vitamins (41%, *n* = 218). Although it was not the focus of this study, it is important to note that health problems can arise from deficiencies and toxicities [6]. For this reason, the most prudent approach is to use supplements formulated specifically for flying foxes, as their nutritional needs differ from those of humans, birds, or other animals [6].

Regarding enclosure conditions, 58% of the flying foxes in our study (*n* = 313) live in adequately sized areas, and most parks maintain facilities with an ideal temperature, except for two parks housing 35 animals where the temperature exceeds the recommended range. Environmental conditions may indirectly influence nutritional imbalances, particularly their effects on social dynamics. Social hierarchy can impact access to essential resources such as food and UVB light, with dominant individuals restricting access for lower-ranked animals, often juveniles. In enclosures that are too small or restrictive, territorial behaviors become more pronounced, increasing the risk of nutritional deficiencies [11,14,32] and potentially contributing to metabolic bone disease (MBD). Although the prevalence of dominance behaviors in zoological parks has yet to be fully assessed, adequate space per individual is crucial in mitigating competition for resources.

Temperature regulation is another key factor, as extreme environmental conditions can impact health. While only very low temperatures affect vitamin D3 synthesis—since heat is necessary to convert pre-vitamin D to cholecalciferol—excessively high temperatures above 305.35 K may induce thermal stress, which, in severe cases, can be fatal [36,37].

All investigated zoological parks have indoor spaces (enclosed areas), and three institutions provide outdoor access. Three parks use ultraviolet lamps, corresponding to 46 flying foxes (9%) with access to artificial light all year round.

Among the parks that are included in our study, metabolic bone disease cases were reported in two, specifically affecting *Pteropus vampyrus*, with ages ranging from one to 15 years and impacting approximately a quarter of the animals in each park. Clinical signs of MBD began developing in the first months of life, suggesting a growth-related disease affecting immature bones, such as rickets [15,17]. Observations revealed that none of the affected animals could fly, with consistent signs including deformities of the long bones in the forearm region (leading to wing bending and arching) and deformation and flexibility of the jaw. These conditions align with rickets and fibrous osteodystrophy (rubber-jaw syndrome) [17,19,32]. In addition, no evident dysfunctions were found in other organs, such as the intestines, liver, kidneys, or parathyroid glands, allowing us to exclude some metabolic bone disease conditions. One of them is primary hyperparathyroidism caused by a parathyroid gland dysfunction (such as adenoma or hyperplasia), responsible for the continuous secretion of parathyroid hormone (PTH), resulting in bone remodeling [15,17]. Secondary renal hyperparathyroidism, triggered by chronic kidney disease, was also ruled out since the affected animals had fully functioning kidneys [18,19]. Screening for hepatic, renal, and intestinal dysfunctions is crucial due to the role of these organs in activating vitamin D3 (primarily in the liver and later in the kidney) and calcium absorption by the intestinal mucosa [19,32,38].

When comparing diets in animals with and without signs of MBD, we observed that animals with the disease consume fewer green vegetables. Green vegetables and fruits contain the highest calcium, phosphorus, magnesium, and vitamin C levels and are essential for proper bone development [33]. Regarding other fruits, no statistically significant differences were observed in animals with signs of MBD. However, flying foxes with MBD tend to eat less purple and orange fruit than animals without the disease.

Regarding primate food, while 59% of healthy animals consume primate food, none of the affected animals consume it. Our data suggest that the intake of these nutrient-rich foods may contribute to the prevention of bone disorders, namely MBD. This food has a high vitamin D3 content, essential for flying foxes living in indoor spaces without access to sunlight. Active vitamin D3 helps maintain calcium homeostasis by facilitating intestinal absorption [23,24].

While supplements are crucial to avoid or mitigate possible deficiencies that might result from a fruit-based diet alone [6], our study suggests that they do not significantly contribute to preventing bone diseases since they are only consumed by 37.6% of healthy individuals. Vitamin and mineral supplements formulated for humans, calcium, vitamin D3, and brewer’s yeast were specifically administered simultaneously by one of the zoological parks with clinically affected animals.

Natural light exposure, particularly in outdoor facilities, appears to be associated with a lower occurrence of clinical signs. An outdoor enclosure provides direct access to sunlight or natural light without barriers, such as glass or plastic, which absorb UVB radiation (wavelength 280–315 nm) and prevent the skin from synthesizing vitamin D3 [39]. As a result, only 173 animals (32%) have access to UVB radiation from the sun and can synthesize vitamin D3 through the cutaneous pathway [37]. However, sunlight exposure alone does not guarantee optimal vitamin D3 synthesis, as it is influenced by factors such as latitude and season [38]. Given that three zoos included in this study are located at approximate latitudes of 30° N, 47° N, and 45° N, vitamin D3 synthesis is expected to be nearly 100% efficient in summer but significantly reduced to 40–60% in spring, 20–45% in autumn, and 10–25% in winter. The highest synthesis rates occur in parks at lower latitudes, while those at higher latitudes experience the greatest seasonal reduction [39].

An alternative approach to ensuring adequate vitamin D3 levels includes the use of artificial UVB-emitting lamps or dietary supplementation [39], but, in our study, exposure to artificial light did not prove to be essential in preventing metabolic bone disease. However, these results must be interpreted cautiously, as most healthy animals lack access to natural and artificial light and, consequently, do not receive UVB radiation, which is essential for the cutaneous synthesis of vitamin D3 [37,39]. Additionally, our data suggest that the majority of animals (94%) could potentially obtain vitamin D3 through their diet, while only 20 animals (3.7%) seemingly lacked access to vitamin D3 from any source, including three individuals diagnosed with metabolic bone disease (MBD). Although vitamin D3 deficiency has been linked to the onset of MBD, potentially influencing intestinal calcium absorption and bone health, the extent to which this factor contributed to disease development in our study remains unclear. Furthermore, our findings raise the possibility that, in these species, dietary intake of vitamin D3 may play a more significant role than UVB radiation exposure in vitamin D metabolism, particularly in flying foxes.

Further research is needed to clarify the role of vitamin D in flying foxes and determine whether dietary intake alone is sufficient or if light activation is necessary, particularly in cases of MBD. Additionally, adequate calcium, phosphorus, magnesium, and vitamin C levels are crucial for bone development. However, discrepancies between predicted and actual dietary values have been reported. Vitamins A, E, and calcium often appear in lower concentrations than expected in consumed diets, while phosphorus, magnesium, and zinc tend to accumulate in leftover food and feces. This suggests possible selective feeding behavior or the absorption of minerals from external sources, such as enclosure materials. Maintaining a well-balanced nutrient intake is essential due to the complex interactions between these elements [7]. These minerals can typically be obtained through diet and supplementation; however, excessive intake may lead to imbalances, potentially resulting in bone disorders due to mineral toxicity [6]. Indeed, one of the zoological parks where this condition was observed provides many supplements, raising the possibility that bone disorders in these animals could be related to mineral toxicity rather than deficiency. Given these findings, it would be advisable to use supplements specifically formulated for flying foxes, ensuring their nutritional requirements are met without exceeding safe intake levels [6].

In addition to issues related to diet and environment, we must also consider the potential role of genetic mutations due to inbreeding or the development of genetic malformations in the etiology of MBD [15]. Osteogenesis imperfecta is one of the genetic malformations that most closely resembles metabolic bone disease. Clinically, it is characterized by pronounced bone fragility, which results in recurrent fractures and skeletal deformities, such as limb bowing. However, its etiology lies in mutations affecting the genes responsible for encoding type I collagen chains, leading to abnormal collagen production and, consequently, impaired bone integrity. This condition can be inherited through either an autosomal dominant or autosomal recessive pattern [15,40]. Although definitive conclusions cannot be drawn due to the lack of genetic testing, the possibility of inbreeding among individuals is strong in one of the affected zoos. In the other zoo, all affected animals underwent genetic testing, and none were found to be related.

While this study provides potential factors influencing metabolic bone disease (MBD) in flying foxes, several limitations must be acknowledged. Besides the low response rate from zoological, the variability in diet, management practices, and environmental conditions across parks complicates the ability to isolate specific causes of the disease. We cannot rule out that, besides vitamin D3, other mechanisms caused by an inappropriate diet for the species may be involved in MBD.

Future research should focus on obtaining a larger sample size and more comprehensive data from a broader range of zoological parks worldwide. Studies should also aim to include detailed genetic analyses to explore the potential role of genetic factors, including inbreeding and mutations, in the development of MBD. In addition, general analyses (of the liver, kidneys, and parathyroid gland) would be important to rule out some metabolic bone disease conditions, as previously mentioned. Furthermore, case studies tracking individual animals over time would provide more robust evidence of the disease’s progression and the effectiveness of various preventive measures.

Despite these limitations, our study highlights several key areas for future investigation.

## 5. Conclusions

This study suggests that vitamin D3 obtained through diet could play a more significant role than that obtained through UVB radiation exposure in preventing MBD in flying foxes, unlike in other bat species. Nonetheless, it is not possible to exclude the environment as a valid source of vitamin D. Our findings also suggest that adopting an appropriate diet specifically tailored to these species could be crucial in preventing MBD and potentially other nutritionally related bone disorders. Moreover, understanding the role of vitamin D3, whether obtained through diet or exposure to UVB light, is essential for formulating effective management strategies. By addressing the gaps identified in our research, future studies could provide a more comprehensive understanding of the disease and contribute to better health and welfare outcomes for flying foxes in human care.

## Figures and Tables

**Table 1:** Other food supplied.

Other Food Items	Number of Animals
Cereal (oatmeal)	6
Primate food	297
Dry feed of frugivorous birds	30
Dry dog food	32

**Table 2:** Cases compatible with metabolic bone disease. Characteristics of the animals and conditions in zoological parks 1 and 2.

Characteristics	Zoological Park 1	Zoological Park 2
Species	*Pteropus vampyrus*	*Pteropus vampyrus*
Number MBD/total animals	6/23 animals	3/12 animals
Onset age	<3 month	<3 month
Diet		
Orange/yellow fruits (%)	37.03	44.5
White fruits (%)	51.87	33.3
Red fruits (%)	3.7	11.1
Purple/blue fruits (%)	3.7	11.1
Green fruits (%)	3.7	Not provided
Other types of food used	Cottage cheese, yogurt, raw eggs, nectar	Not provided
Nutritional supplements	Calcium, vitamin D3, yeast, human supplements	Calcium
Nutritional plan	Yes	Yes
Enclosures		
Dimensions	Not ideal	Ideal
Temperature	Not ideal	Not ideal
Natural light	No	No
Artificial light	Yes	No
Outcome	All alive	2/3 Euthanized

## Data Availability

The original contributions presented in the study are included in the article/Supplementary Material; further inquiries can be directed at the corresponding author.

## Supplementary Materials

The following supporting information can be downloaded at: https://www.mdpi.com/article/10.3390/vetsci12030271/s1, File S1: Survey.
