# Supplementary material for: Metabolic Bone Disease in Captive Flying Foxes: A Comprehensive Survey Across Zoological Parks
Source: Vet Sci. 2025 Mar 13;12(3):271. doi: 10.3390/vetsci12030271 (PMC11946488; doi:10.3390/vetsci12030271)
Supplement: Supplementary file 1 [file vetsci-12-00271-s001.zip › vetsci-3478777-supplementary.pdf]

Supplementary Files

Survey

Title: Prevalence study of metabolic bone disease in captive flying foxes

Introdution: This survey is being carried out as part of an integrated Master's degree in Veterinary Medicine and is incorporated into a project on the prevalence of metabolic bone disease in bats (D. Faim, I. Pires, A. Weissenbache, 2023). My goal with this project is to obtain more accurate information about the metabolic bone disease in flying foxes, namely what are the possible causes of its ocurrence. To obtain these results I would like to ask you to answer this survey as objectively and completely as possible, since the variables of the study have a crucial role in the discussion of the results. Finally, the relevance of this project would be to study solutions that could avoid this problem in future generations and thus improve the health of the animals and their welfare. The results will only be used for academic/scientific purposes and the survey is anonymous. If you have any questions or additional interest in this project please contact me at dianasofaim@gmail.com

Questions:

Please answer the following questions

1. What is the name of the zoological park/rehabilitation center? *
2. What is the geographic location of the zoological park/rehabilitation center? *
3. Which species of flying foxes do you have? *

Regarding feeding:

4. What is the food provided? (Select one or more options): *

(fruit/vegetables, nectar and pollen from flowers, animal products)

5. In the case of fruit/vegetables, list which ones and their proportion:
6. In the case of nectar from flowers, list which ones and their proportion:
7. In the case of animal products, list which ones and their proportion:
8. Do you provide supplements in addition to food? Yes or no *
9. If yes, which ones and what amount?
10. Do you follow a menu or a nutritional plan as a routine? Yes or no *

Regarding facilities:

11. How many square meters per animal? *
12. Is it na indoor or outdoor space, or both? Indoor/outdoor/both *
13. What is the temperature of the installations? *
14. Do they have access to natural sunlight? Yes/no*

15. If yes, how many hours per day on average, in each season?

16. Do they have access to ultraviolet light (artificial)? Yes/no *

17. If yes, how many hours per day?

18. If yes, how many lamps?

19. If yes, what is the range of the lamps (number of meters reached by the radiation)?

Regarding the state of health

20. At this zoological park, do any flying foxes show signs of metabolic bone disease? (exemples: bone deformities , swollen/thickened limb bones, soft/pliable or deformed jaw, fractures, generalized weakness , inability to grow, inability to fly, reluctance to move)? Yes/No *

If yes, please answer the following questions

21. Which signs of metabolic bone disease do flying foxes show? (Select one or more options): bone deformities , swollen/thickened limb bones, soft/pliable or deformed jaw, fractures, generalized weakness , inability to grow, inability to fly, reluctance to move, another one)

22. How many animals present these signs?

23. How old are they?

24. In case of being females, any of them are pregnant or lactating?

25. Do they have precedents with the same problem?

26. If yes, is the male or is the female or both? Male/female/both

27. In addition to metabolic bone disease, do you have any disease or dysfunction in the following organs? Select one option or several options (parathyroid gland, thyroid, liver, kidney,intestine, none)

28. In case of having animals with metabolic bone disease (deformation of bones), would you be available to perform biochemical analysis and an X-ray, in order to get more results ? Yes/no *

29. In case of having records of biochemical analysis (with particular interest of vitamin D, calcium and phosphorus), and X-rays of previous years, would you mind to provide them, in order to collect as many results as possible? Yes/no *

Thank you very much for your cooperation in answering this survey, it will provide valuable data for the success of this project. In case you have given an affirmative answer to the last two question, we will contact you again for further indications, thank you!
